# Solubilization and purification of recombinant modified C-reactive protein from inclusion bodies using reversible anhydride modification

**DOI:** 10.1007/s41048-015-0003-2

**Published:** 2015-07-18

**Authors:** Lawrence A. Potempa, Zhen-Yu Yao, Shang-Rong Ji, János G. Filep, Yi Wu

**Affiliations:** Roosevelt University College of Pharmacy, Schaumburg, IL USA; MOE Key Laboratory of Cell Activities and Stress Adaptations, School of Life Sciences, Lanzhou University, Lanzhou, 730000 People’s Republic of China; Key Laboratory of Preclinical Study for New Drugs of Gansu Province, Lanzhou University, Lanzhou, 730000 People’s Republic of China; Research Center, Maisonneuve-Rosemont Hospital, University of Montréal, Montréal, QC Canada

**Keywords:** Recombinant modified C-reactive protein, Inclusion bodies, Citraconic anhydride

## Abstract

The precise function of C-reactive protein (CRP) as a regulator of inflammation in health and disease continues to evolve. The true understanding of its role in host defense responses has been hampered by numerous reports of comparable systems with contradictory interpretations of CRP as a stimulator, suppressor, or benign contributor to such processes. These discrepancies may be explained in part by the existence of a naturally occurring CRP isoform, termed modified CRP (i.e., mCRP), that is expressed when CRP subunits are dissociated into monomeric structures. The free mCRP subunit undergoes a non-proteolytic conformational change that has unique solubility, antigenicity, and bioactivity compared to the subunits that remain associated in the native, pentameric CRP molecule (i.e., pCRP). As specific reagents have been developed to identify and quantify mCRP, it has become apparent that this isoform can be formed spontaneously in calcium-free solutions. Furthermore, mCRP can be expressed on perturbed cell membranes with as little as 24–48 h incubation in tissue culture. Because mCRP has the same size as pCRP subunits as evaluated by SDS-PAGE, its presence in a pCRP reagent would not be apparent using this technique to evaluate purity. Finally, because many antibody reagents purported to be specific for “CRP” contains some, or substantial specificity to mCRP, antigen-detection techniques using such reagents may fail to distinguish the specific CRP isoform detected. All these caveats concerning CRP structures and measurements suggest that the aforementioned contradictory studies may reflect to some extent on distinctive bioactivities of mCRP rather than on pCRP. To provide a reliable, abundant supply of mCRP for separate and comparable studies, a recombinant protein was engineered and expressed in *E. coli* (i.e., recombinant mCRP or r_m_CRP). Synthesized protein was produced as inclusion bodies which proved difficult to solubilize for purification and characterization. Herein, we describe a method using anhydride reagents to effectively solubilize r_m_CRP and allow for chromatographic purification in high yield and free of contaminating endotoxin. Furthermore, the purified r_m_CRP reagent represents an excellent comparable protein to the biologically produced mCRP and as a distinctive reagent from pCRP. Deciphering the true function of CRP in both health and disease requires a knowledge, understanding, and reliable supply of each of its structures so to define the distinctive effects of each on the body’s response to tissue damaging events.

## Introduction

C-reactive protein (CRP) is generally regarded as one of, if not the pre-eminent protein of the early acute phase response. Its concentration in serum is a valuable diagnostic tool as an index of inflammatory tissue damage, rising from a baseline of 1–3 μg/mL to 100 s of μg/mL within 8~72 h of any stress that affects tissue integrity (Kushner 1982; Morley and Kushner 1982; Gewurz et al. 1995; Gabay and Hushner 1999; Black et al. 2004). As the inflammatory response gets resolved, serum CRP levels quickly return to baseline. Since its fractional catabolic rate is independent of its serum concentration and its clearance is independent of the stage and type of disease that elicit its response, the exact relevance of blood-borne CRP to the body’s response to disease remains unknown. Persistently elevated CRP concentration reflects on an increased rate of hepatic synthesis and release and is generally regarded as a reliable indicator of low-level chronic inflammation occurring somewhere in the individual (Macintyre et al. 1982, 1985; Vigushin et al. 1993).

Because of the definite association of serum CRP levels and inflammation, it has long been perceived as some kind of regulator of this process. Efforts to precisely define its biological role have identified various ligands, tissue distribution, effects on cellular and humoral effector responses, and relevance as a predictive factor for development of diseases (Ridker et al. 1997; Marnell et al. 2005; Ma et al. 2013; DuClos 2013; Agrawal et al. 2014). Frustratingly, many reports are in direct contradiction, and a consensus agreement for CRP’s exact biological role remains elusive (Paul et al. 2004; Hirschfield et al. 2005; Kovacs et al. 2007; Teupser et al. 2011).

Our efforts to understand the bioimmunological role for CRP have focused on carefully examining its higher order structure (i.e., secondary, tertiary, and quaternary) and the changes to subunit structure when dissociated from the CRP pentameric disk. We discovered that when pCRP subunits are separated, they undergo a notable conformational change which markedly affects CRP solubility, antigenicity, biodistribution, cellular, and humoral reactivities (Potempa et al. 1983, 1987; Ying et al. 1989; Schwedler et al. 2006; Wu et al. 2007; Wang et al. 2011). Furthermore, we found that these structural changes can occur undetected and to various extents in CRP test reagents and CRP experimental systems (Potempa et al. 1987; Khreiss et al. 2004a, b; Ji et al. 2007). Of significance, if mCRP generation was not recognized and controlled for, it may have been present in CRP reagents used by various investigators, influencing experimental observations and confounding conclusions reached regarding CRP’s exact biological role.

To more carefully study the free subunit isoform of CRP, designated as monomeric, modified CRP (i.e., mCRP), a recombinant protein reagent was engineered and expressed in *Escherichia coli*. While significant amounts of protein were produced after induction, the inherent insolubility of the manufactured recombinant analog of mCRP, (termed r_m_CRP), was manifest as difficult-to-process inclusion bodies. Herein we describe a method to solubilize and purify r_m_CRP and present evidence for its usefulness as a comparable and effective reagent for examining the differential characteristics of mCRP.

We describe a simple, reversible method to solubilize inclusion body aggregated r_m_CRP using citraconic anhydride. The solubilized protein was efficiently and effectively processed by hydrophobic and anion exchange chromatographic methods to produce highly purified, endotoxin-free protein. This purified protein is structurally, antigenically, and functionally analogous to biologically generated mCRP and represents a useful and advantageous reagent for the expanded study of the conformationally distinctive CRP-free subunit (Zouki et al. 2001; Khreiss et al. 2002; Ji et al. 2006a, b).

## Results

### Preparation of biological modified (mCRP) from native (pentameric) CRP (pCRP)

Biological pentameric CRP (pCRP) was purified from human pleural or ascites fluids using calcium-dependent affinity chromatography to phosphorylcholine-substituted agarose resin essentially as described (Potempa et al. 1987). Affinity bound protein was eluted with citrate and was subsequently processed on Q-Sepharose anion exchange to reduce endotoxin contamination and improve purity. Eluted protein was immediately dialyzed into 25 mmol/L Tris–HCl, 0.15 mol/L NaCl (pH 7.4) containing 2 mmol/L CaCl_2_, sterile filtered and stored at 4 °C. It is important to note that storage of native CRP at 4 °C without calcium, or in the presence of cheating reagents, will result in a slow, spontaneous conversion of pCRP into the mCRP conformer. Storage of pCRP at temperatures below freezing is not recommended as irreversible alterations in pentameric CRP protein conformation have been observed.

**Fig. 1:**
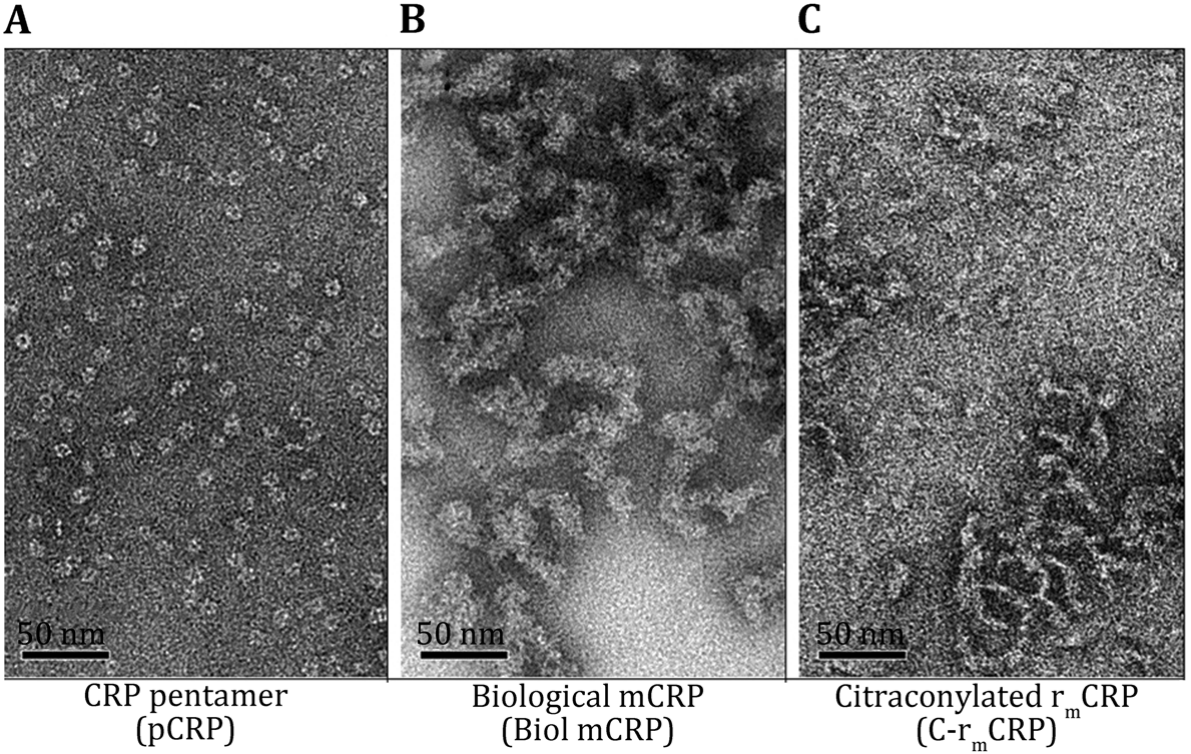
Visualization of pCRP, biological mCRP, and C-r_m_CRP. Visualizing the distinct structural differences between the pCRP pentameric disk conformer and the subunit mCRP fibrous-like conformer, whether produced from the pCRP protein (bio-mCRP) or by recombinant expression (C-r_m_CRP). A 2 μL droplet of sample was added to a freshly glow-discharged carbon-coated 300 mesh copper EM grid for 10 s followed by staining with 1 % PTA for 60 s. Grids were observed in a Tecnai G20 (FEI) 200 kV EM. All magnifications are 50,000×. Pentametic CRP (pCRP) **A** is seen to exist as cyclic pentameric discoids of tightly packed subunits surrounding a central void with an average diameter of 10.42 ± 0.08 nm (*n* = 200). Biological mCRP (Biol-mCRP) **B** was produced from pCRP using urea chelation (see Method). It is seen to exist as short, fat fibrous aggregates with an average diameter of 14.45 ± 0.33 nm (*n* = 20). Citraconylated, recombinant modified CRP (C-r_m_CRP) **c** exists primarily as short, thinner fibers. An average diameter of 9.92 ± 0.24 nm (*n* = 25). The scale bars represent 50 nm

mCRP was prepared from purified pCRP pentamer using urea chelation (i.e. biological mCRP) (Potempa et al. 1983, 1987). Specifically, ultrapure urea was dissolved to 8 mol/L into a pCRP solution, and EDTA was added to 10 mmol/L. The solution was incubated for 1 h at 37 °C and then exhaustively dialyzed into 25 mmol/L Tris–HCl, (pH 8.3). Under these conditions, the maximum solubility of mCRP was found to be 0.6 mg/mL. The addition of >0.1 mol/L sodium or potassium chloride, or 1 mmol/L CaCl_2_, MnCl_2_, NiCl_2_, or ZnCl_2_, 3.5 mmol/L MgCl_2_, or 0.1 mmol/L FeCl_2_ resulted in the immediate self-aggregation of mCRP into an opalescent suspension (i.e., reduced aqueous solubility). By electron microscopic analysis, pCRP is seen as a tightly compacted pentameric disk of five globular subunits surrounding an annular void in agreement with the protein structure defined by crystallization analyses (Shrive et al. 1996) (Fig. 1A). Biological mCRP is seen to exist as short but fat fiber aggregates that do not appear to be dense structures but rather loose associated short rod-like structures (Fig. 1B). mCRP molecules in the absence of added salt or in the presence of membranes were seen to associate into diffuse matrix-like structures very distinct from the annular pentameric pCRP disk. In the presence of salt, the mCRP diffuse aggregates are seen to condense into large homogeneous electron dense clusters (Ji et al. 2007; Motie et al. 1996).

### Purification of cys-mutated r_m_CRP from inclusion bodies

The *E. coli* expressed r_m_CRP protein deposited as highly aggregated, insoluble inclusion bodies. Numerous solubilization methods including up to 8 mol/L urea, up to 6 mol/L guanidinium hydrochloride, low and high ionic strength salt solutions, addition of arginine or protic solvents such as n-propyl alcohol, addition of ionic (e.g., SDS) and non-ionic (e.g., Triton X 100) detergents at various concentrations, and increasing pH values > 12.0 were tried with limited or no success. Each method, including different combinations of each, was insufficient to allow for chromatographic purification, was found to interfere with biochemical separation methodologies, or was found to be too harsh and damaging to protein integrity.

The dilemma of how to solubilize the cys-mutated r_m_CRP was solved by a serendipitous observation on the solubility of biological mCRP while investigating the amino acid residues in the CRP sequence/structure that contributed to mCRP binding activity for immune complexes (Motie et al. 1996). Various site-specific modification reactions were performed on biological mCRP to block or alter selected amino acid R groups prior to performing binding assays. A brief summary of reagents used and the general effects on immune complex binding is shown in Table 1.

**Table 1:** Group-specific modification reagents used to affect mCRP- aggregated IgG interactions

Amino acid group targeted	Group modification reagent	Reaction of aggregated IgG with group-modified mCRP	Reaction of mCRP with group-modified aggregated IgG
Epsilon amino group of lysine	NHS biotin	Strong binding	*No binding*
Guanidino group of arginine	*p*-hydroxyphenylglyoxal	Strong binding	Strong binding
Indole ring of tryptophan	BNPS-Skatole	*Decreased binding*	Strong binding
Phenyl ring of Tyrosine	Tetranitromethane	Strong binding	*Decreased binding*
Carboxyl groups of aspartic and glutamic acid	Diazo nor-leucine methylester	*Decreased binding*	Strong binding
Sulfhydryl group of cysteine	*N*-ethyl maleimide	Strong binding	Strong binding
Thiomethyl group of Methionine	Hydrogen peroxide	Strong binding	Strong binding

As summarized from studies reported by Motie et al. (1996)

These studies established that anionic groups and tryptophan residues on the mCRP structure are required for binding aggregated (complexed) IgG structures. More relevant to this report, it was noted that modification of primary amine residues with various anhydride reagents notably increased mCRP solubility. A systematic evaluation of various anhydride reagents (i.e., succinyl, maleic, and citraconic anhydrides) all effectively increased the solubility of mCRP. Furthermore, even though anhydride modification resulted in changing the positive charge on primary amine residues to negatively charged carboxyl groups, such changes were not detrimental to the studied mCRP binding activities, nor its antigenicity using monoclonal antibodies specific for the mCRP isoform (Ying et al. 1989, 1992). Because of the desire to allow for facilitated removal of the acyl groups for directed studies, and because citraconic anhydride is supplied as a liquid reagent that can be easily aliquoted, it was chosen as the preferred solubilizing agent for processing inclusion body aggregated cys-mutated r_m_CRP.

### Solubilization of inclusion body protein using citraconic anhydride

To approximately 20 mL inclusion body at 60 mg/mL protein, approximately 150 mL 0.1 mol/L NaHCO_3_ (pH 9) was added and stirred to form a homogeneous suspension. While stirring, a 200 μL aliquot of citraconyl anhydride was added to the suspension, and the pH was continuously monitored. At these slightly basic conditions, primary amines are nucleophilic and will readily react with the added anhydride reagent. As the reaction proceeded, the pH of the solution dropped, slowing the reaction rate. To maintain high efficiency of acylation, the suspension pH was continually monitored so to maintain a reaction pH at 9.0 by adding drops of 1 N NaOH. As anhydride reagent was depleted, the pH change slowed or stopped. Additional aliquots of citraconic acid (up to 10 for the system described here) were added and the cycle repeated. While all IB protein was modified to some extent, not all precipitated materials in the inclusion body suspension immediately solubilized at this step.

After the pH stabilized with the final addition of anhydride reagent, the inclusion body slurry was centrifuged at 3000×*g* at 4 °C for 30 min. Care was taken not to pellet the protein at such high centrifugal forces that would cause the protein to compact, complicating recovery of the precipitate for base-induced solubilization (see below). The soluble supernate (containing citraconylated cys-mutated r_m_CRP (i.e., C-r_m_CRP) and various other inclusion body contaminants) was collected and was immediately passed on a Phenyl Sepharose hydrophobic adsorption column. The non-solubilized citraconic anhydride-treated inclusion body pellet that was collected by centrifugation, resuspended in 0.1 mol/L NaHCO_3_ (pH 8.5) and solubilized by a brief exposure to pH > 12.0 (using 5 N NaOH). After the base-solubilized protein clarified, the pH was quickly returned to pH 8.5 by the addition of 6 N HCl. This base-solubilized sample remained soluble indicating it too has been sufficiently modified by acylation to enhance inclusion body solubilization. Approximate half of the cys-mutated r_m_CRP in the starting inclusion body sample was found to directly solubilize using the method described here, and half required a brief exposure to high pHs to achieve solubilization.

### Phenyl Sepharose hydrophobic adsorption chromatography

Phenyl Sepharose (high substitution, fast flow) hydrophobic adsorption resin (350 mL in a 3.0 × 50 cm column) was equilibrated in 0.1 N NaHCO_3_ (pH 8.5). Solubilized C-r_m_CRP, containing approximately 600 mg protein, was directly applied at a flow rate of 4 mL/min. Non-bound material was collected and found to be essentially protein free, containing cell breakage debris that had significant absorbance at 260 nm and minimal absorbance at 280 nm.

After extensive washing with 0.1 mol/L NaHCO_3_, the resin-adsorbed protein was further washed and equilibrated with 0.1 mol/L Na/K Phosphate buffer (pH 7.4). The ratio of sodium to potassium ions in this buffer was achieved by mixing dibasic sodium phosphate and monobasic potassium phosphate until the desired pH of 7.4 was reached.

**Fig. 2:**
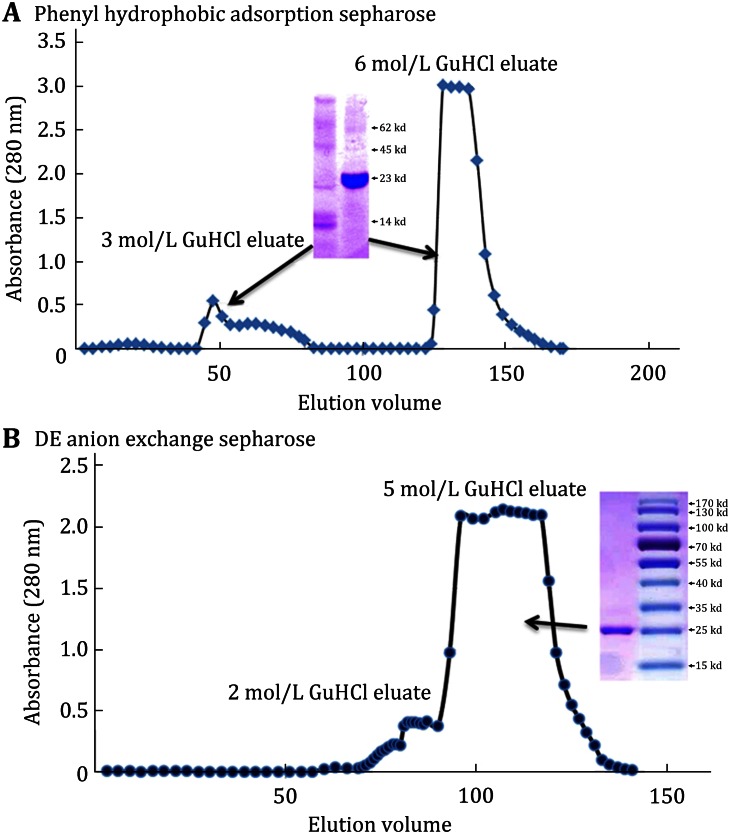
Chromatographic elution profiles of C-r_m_CRP from inclusion bodies. Demonstration of representative elution profiles of citraconylated recombinant mCRP from inclusion body preparations. **A** Phenyl Sepharose (high substitution, fast flow) hydrophobic adsorption resin (3.0 × 50 cm) was equilibrated in 0.1 N NaHCO_3_ (pH 8.5) and Citraconylated Inclusion bodies of *E. coli* expressed r_m_CRP, containing approximately 600 mg protein, was applied at a flow rate of 4 mL/min. After washing and equilibration in neutral phosphate buffer, protein fragments and aggregates eluted with passage of 3 mol/L GuHCl (see representative SDS-PAGE analysis in a *inset*). C-r_m_CRP (SDS-PAGE app Mw of 23 kD) eluted with passage of 6 mol/L GuHCl. **B** After collection and processing (See Methods), the eluted C-r_m_CRP was further processed on DE Sepharose fast flow anion exchange resin (3.0 × 50 cm column), remaining bound using 2 mol/L GuHCl and eluting using 5 mol/L GuHCl. Purified C-r_m_CRP calculated Mw = 23,488 (B *inset*)

Loosely adsorbed contaminants were removed by passing 1 mol/L NaCl in 0.1 mol/L Na/KPO_4_ (pH 7.4). Additional contaminants and C-r_m_CRP fragments and aggregates were eluted using 3 mol/L Guanidinium HCl (GuHCl) in 0.1 mol/L Na/KPO_4_ (pH 7.4). This was followed by passage of 6 mol/L GuHCl in 0.1 mol/L Na/KPO_4_ (pH 7.4), where C-r_m_CRP effectively eluted (Fig. 2A).

The 6 mol/L GuHCl eluted pool was simply diluted 1 ➔ 5 with RO water. This method reduced the effective GuHCl concentration to ~1 mol/L and resulted in immediate precipitation of all C-r_m_CRP in the eluted pool which was clearly visible. The diluted precipitated eluate was allowed to equilibrate at 4 °C overnight with gentle stirring. The precipitate was then collected as described above by centrifugation at 3000×*g* at 4 °C for 30 min. The diluted GuHCl supernatant was decanted and discarded. The centrifuged precipitate was gently resuspended in 0.1 mol/L NaHCO_3_ (pH 8.5) and then resolubilized by slow addition of 5 N NaOH until the pH was raised above 12.0. Once the protein solution clarified, the pH was quickly brought back down to pH 8.5 with the addition of 6 N HCl under which conditions the protein remained soluble. Of note, exposure of the protein to these high pH values was limited to as short a time as possible to avoid base-induced protein modification.

### DE Sepharose anion exchange chromatography

DE Sepharose fast flow anion exchange resin (350 mL in a 3.0 50 cm column) was equilibrated in 0.1 N NaHCO_3_ (pH 8.5). Of note, use of the stronger anion exchange Q-Sepharose resin is not recommended for the purification of C-r_m_CRP as increased levels of fragmentation were noted in final eluted protein.

The resolubilized Phenyl Sepharose 6 mol/L GuHCl eluate was directly applied and bound to the DE Sepharose resin. The column was exhaustively washed with 0.1 mol/L NaHCO_3_ (pH 8.5), which was then equilibrated in 0.1 mol/L Na/KPO_4_ buffer (pH 7.4). Loosely bound contaminants were removed by washing with 1 mol/L NaCl in 0.1 mol/L Na/K PO_4_ (pH 7.4).

Protein fragments and other contaminants were eluted with 2 mol/L GuHCl in 0.1 mol/L Na/K Phosphate buffer (pH 7.4) followed by elution of the main protein peak with 5 mol/L GuHCl in Na/KPO_4_ buffer (Fig. 2B).

The eluted protein was processed exactly as described above with 6 mol/L GuHCl Phenyl Sepharose eluate. The pooled peak was diluted 1➔5 with RO water to reduce the GuHCl concentration, which led to protein self-aggregation. Aggregates were collected by centrifugation, resuspended in 0.1 mol/L NaHCO_3_, and solubilized by adding 5 N NaOH until the solution clarified. The resolubilized protein was quickly neutralized and was exhaustively dialyzed into a final buffer of choice—most generally into 25 mmol/L Na phosphate buffer containing 0.15 mol/L NaCl (pH 7.4) (i.e., 25 mmol/L NaPBS).

The final processed protein pool was sterile filtered through 0.2 μm membranes. Protein concentration was measured by BCA assay and by absorbance at 280 nm, using Beer’s law. SDS-PAGE analysis (see inset, Fig. 2B), indicated that the C-r_m_CRP was >95 % pure with an apparent molecular weight of 23,488 daltons.

Unpurified, non-citraconylated starting inclusion body protein (i.e., crude inclusion body protein) was solubilized using SDS detergent and run on SDS-PAGE gels for direct comparison to the purified C-r_m_CRP protein. Crude inclusion body protein was predominantly r_m_CRP (minimal contaminating bands were also observed) and migrated with an apparent molecular weight of 22,827 (data not shown). The difference in size between the Citraconylated r_m_CRP and the non-citraconated r_m_CRP (i.e., 661 daltons) is consistent with substitution of 6 citraconyl groups on the derivatized protein (based on a molar weight of 112 daltons for each citraconyl group). These data are consistent with calculations of molar substitution based on the anhydride reagent extinction coefficient and absorbance measurements at 250 nm (see Methods).

The recovered C-r_m_CRP retained a high level of solubility even in saline-based buffers (up to ~6.5 mg/mL). The fact that the highly concentrated protein could pass through 0.2 μm Acrodiscs suggest that the protein is not forming microaggregates that would otherwise clog filter membranes.

### Procedure for removing citraconyl groups (if desired)

Citraconyl groups are removed from the C-r_m_CRP by simple dialysis into 0.05 mol/L Citrate, pH 3.5, for 2 h at RT. The purified r_m_CRP protein will self-aggregate and form a suspension which remains when the decitraconylated protein is re-equilibrated into saline-based buffers of neutral pH. The opalescent suspension is visually similar to that observed for biological mCRP when urea-chelated mCRP is dialyzed into saline-based buffers to remove urea.

### SDS-PAGE and western blot analysis

Western blot analysis using monoclonal antibodies specific for the mCRP isoform (Ying et al. 1989, 1992), verifying that the main isolated protein band was >95 % pure and consistent with a free CRP subunit size (i.e., 23 kDa). It exhibited antigenicity similar to the mCRP and not pCRP antigen (data not shown).

### Amino acid composition

Purified non-citraconylated r_m_CRP was analyzed for amino acid composition by standard analysis and was found to contain the same number of amino acid residues per mole of protein as the biologically purified CRP subunit. Eleven alanine residues were quantified per mole of recombinant CRP compared to 9 per mole of mCRP, and two cysteine residues were quantified per mole of mCRP while none were detected in r_m_CRP. Three methionine resides were quantified per mole of r_m_CRP compared to two in the biological CRP subunit. These results corroborate the changes engineered in the recombinant CRP subunit and verifying the expressed protein is in other ways identical to the biologically derived CRP molecule. These procedures were performed under contract by Analytical Biotechnology Services, Boston, MA.

### N-terminal sequence analysis of r_m_CRP

Non-citraconylated r_m_CRP was bound to a Biobrene-treated glass filter, and the N-terminal residues were cleaved using phenyl isothiocyanate (PITC) in a pulsed liquid sequencer with an in-line Phenyl-thiohydantoin amino acid analyzer. The cycle was repeated 15 times to determine the N-terminal sequence of the r_m_CRP protein. The sequence determined—MQTDMSRKAFVFPKE—exactly corresponds to the sequence established for the CRP subunit (Shrive et al. 1996). This analysis was performed under contract by Analytical Biotechnology Services, Boston, MA.

### Peptide mapping and mass spectroscopy of r_m_CRP

Non-citraconylated r_m_CRP and mCRP were solubilized in 8 mol/L urea and were treated with dithiothreitol and iodoacetate. The samples were diluted four-fold and were digested with endoproteinase Lyc-C. Digests were separated on reverse phase C-18 HPLC resin using an acetonitrile gradient in the presence of TFA. Separated peptides were analyzed for mass using electrospray mass spectroscopy.

Lys-C digested r_m_CRP produced peptide maps that matched closely to those maps generated with the biological CRP subunit. No major amino acid substitutions were present in the recombinant product that would results in segments of the protein that are different from segments of the biological CRP subunit. These analyses were performed under contract by the Protein and Carbohydrate Structure Facility, Ann Arbor, MI.

### Serum protein electrophoresis of pCRP, mCRP and citraconylated r_m_CRP

**Fig. 3:**
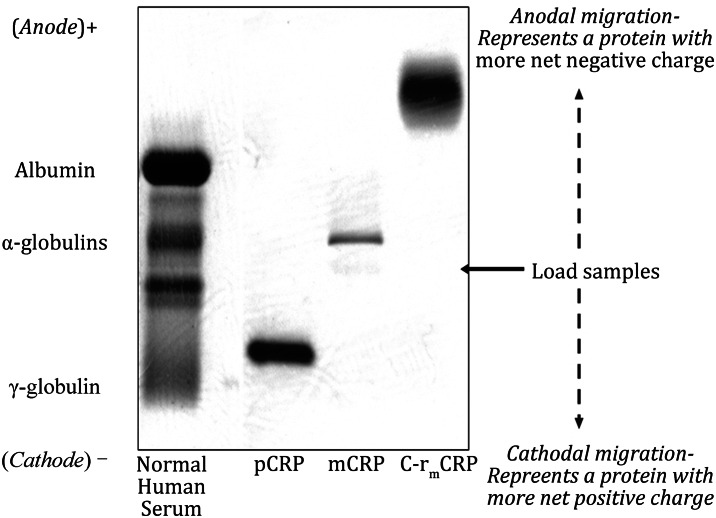
Serum protein Electrophoresis of pCRP, mCRP, and C-rmCRP. Comparing the electrophoretic mobility of mCRP produced from the pCRP after urea chelation to pCRP mobility. The conversion of pCRP to mCRP increased the electrophoretic mobility of CRP from gamma to alpha. The effect of solubilizing recombinant mCRP with citraconic anhydride (C-r_m_CRP), which changes primary amine residues to carboxyl residues is shown for comparison. The anionized r_m_CRP displays rapid pre-albumin mobility

CRP pentamer (pCRP) migrates as a gamma globulin by Serum Protein electrophoresis, having an apparent isoelectric point of 6.3 ± 0.2 (Laurent et al. 1983; Potempa et al. 1983). Based on its primary amino acid sequence, the calculated isoelectric point for CRP is 5.3, which would be expected to have alpha rather than gamma electrophoretic mobility (Potempa et al. 1983). When pCRP is converted into the mCRP, the mCRP is seen to now migrate toward the anode in the predicted alpha zone (Fig. 3). Due to the added negative charges after r_m_CRP is acylated with citaconic anhydride, the anionized protein is seem to migrate with pre-albumin anodal electrophoretic mobility.

### Antigenicity comparisons of native CRP, mCRP, and all forms of r_m_CRP

**Fig. 4:**
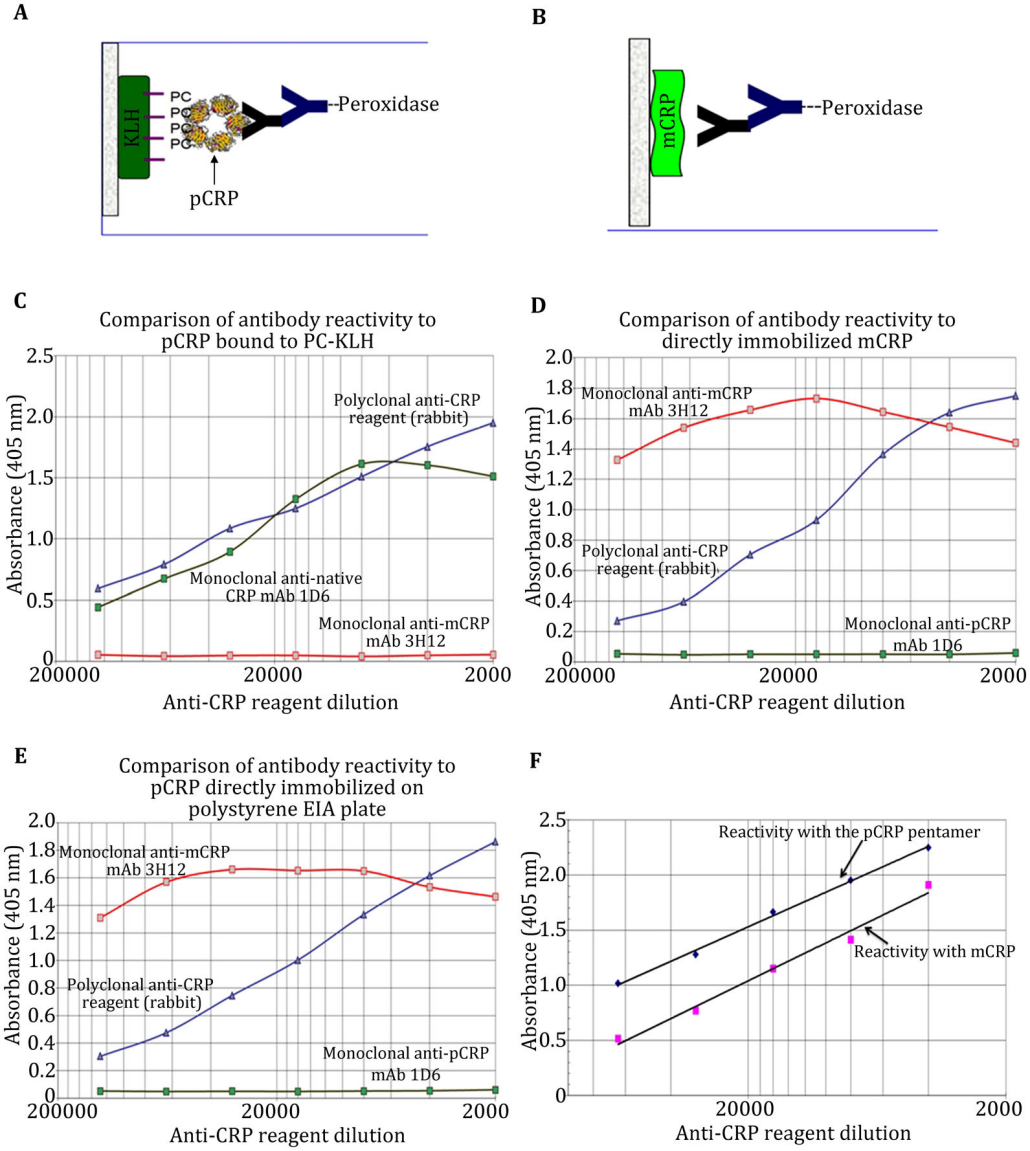
Antigenic comparison of pCRP, mCRP, and C-r_m_CRP. Demonstration of dual CRP specificities in a polyclonal anti-CRP reagent. Evaluation of a polyclonal anti-CRP reagent for binding specificity to pCRP and mCRP antigens. ELISA analyses were performed using various primary coat concentrations and various dilutions of defined monoclonal anti-CRP reagents (Ying et al. 1989, 1992) and a representative polyclonal anti-CRP reagent. In this analysis, results are shown for an IgG fraction anti-Human C-reactive protein antiserum produced in rabbits (see Methods). **A** depicts the ELISA construct to detect pCRP. Phosphorylcholine-substituted KLH (PC-KLH) was first immobilized on ELISA polystyrene surfaces before pCRP was captured as a function of calcium. **B** depicts the ELISA construct to detect mCRP. mCRP was produced from pCRP by urea chelation and was directly immobilized onto the plate surface. Of note, direct immobilization of the CRP pentamer onto the polystyrene surface will result in loss of pCRP structure and expression of mCRP structure (see below, E). **C** demonstrates the antigenic reactivity of anti-pCRP monoclonal antibody 1D6 for PC-captured pCRP and directly comparable reactivity of the rabbit anti-CRP polyclonal reagent. The anti-mCRP monoclonal antibody 3H12 shows no reactivity for PC-captured pCRP antigen. **D** demonstrates the antigenic reactivity of these same antibody reagents at the same tested concentrations for the mCRP antigen. These results show that while monoclonal anti-mCRP 3H12 now shows significant and distinctive reactivity, anti-pCRP monoclonal antibody1D6 is non-reactive. The rabbit anti-CRP polyclonal reagent, however, shows substantial reactivity for the mCRP antigen. **E** demonstrates the effect of directly immobilizing native CRP on the plate surface, demonstrating that the pCRP antigenicity is lost and the mCRP antigenicity is expressed when CRP is adsorbed onto the plastic surface. **F** shows a least-squares analysis comparing the relative reactivity of the rabbit anti-CRP polyclonal reagent used herein. Equivalent concentrations of either pCRP and mCRP antigens were immobilized, and various dilutions of polyclonal reagents were reacted under equivalent experimental conditions. Quantified reactivity was plotted as a function of antiserum dilution, and the level of reactivity for each antigen was normalized using the regression analyses. Results show the rabbit anti-CRP reagent tested expressed both pCRP and mCRP antigenic specificity and that the mCRP reactivity represented between 25 and 33 % the pCRP antigenicity measured. This analysis was repeated multiple times using multiple levels of immobilized antigens using various other commercially available anti-CRP reagents made in different species, and in anti-CRP polyclonal antiserum made in our laboratory. The results shown here are representative of all polyclonal anti-CRP reagents tested

Various monoclonal antibodies have been developed, which specifically react with the pCRP and mCRP isoforms (Ying et al. 1989). Of note, direct adsorption of pCRP onto plastic surfaces will result in the loss of pCRP antigenicity and the expression of mCRP antigenicity. Hence, to monitor pCRP antigenicity in solid phase assays, pCRP must be captured onto a specific ligand that is directly adsorbed to the plastic surface (e.g., using CRP calcium-dependent specificity for phosphorylcholine (substituted on keyhole limpet hemocyanin (PC-KLH)), or to antibodies that are specific for native CRP) (the construct is shown in Fig. 4A).

PCRP and mCRP antigens are clearly differentiated using these monoclonal antibodies. Anti pCRP mAb 1D6 strongly reacted with captured pCRP and not with mCRP (Fig. 4C) while the mAb 3H12 strongly reacted with mCRP and not with captured pCRP (Fig. 4D). Using identical assays as just described, a representative polyclonal rabbit anti-human CRP reagent was screened for specificity to both pCRP and mCRP antigens. The reagent was found to have strong reactivity to both isoforms (Fig. 4C, D). Similar assays have been performed using other polyclonal anti-CRP reagents (e.g., produced in goat, sheep, etc.) with similar results, indicating reagents labeled as “anti-CRP” have a high probability of containing dual specificity to both the pCRP and the mCRP antigen (Samberg et al. 1988). Furthermore, the widely used anti-CRP monoclonal reagent known as Clone 8 (Sigma-Aldrich), was also screened using these assays and found to be predominantly specific for the mCRP and not the pCRP antigen (Schwedler et al. 2003).

Both crude and purified C-r_m_CRP produced in *E. coli* express mCRP and not pCRP epitopes. mAbs directed to the C-terminal octapeptide of the CRP subunit, which is only expressed in the mCRP isoform (Ying et al. 1992) react with the same specificity and affinity to mCRP, r_m_CRP, and to alkylated or de-alkylated forms of r_m_CRP. A mAb that reacts with a sequence of mCRP that involves residue cysteine 36 (i.e., mAb 8C10) shows lesser reactivity with all r_m_CRP proteins, suggesting that the C36A, C97A mutations that remove the CRP subunit disulfide bond, do influence expression of the epitope recognized by this mAb. All other mAbs tested showed comparable reactivity of mCRP and r_m_CRP. Hence, based on sequence, solubility, and antigenicity characteristics, recombinant mCRP is directly comparable to biological mCRP.

When the polyclonal reagent is titered on various levels of immobilized antigens and its relative reactivity for pCRP and mCRP is compared, a linear regression analysis allows for an estimation of the percent of mCRP specificity in the anti-CRP polyclonal reagent. Using this analysis (Fig. 4F) the representative rabbit anti-CRP polyclonal reagent shown herein was estimated to have from 25 to 33 % specificity for the mCRP antigen.

### Higher order structure of CRP, mCRP, and C-r_m_CRP

CRP is a highly soluble protein comprised five globular subunits arranged in cyclic symmetry. Its three-dimensional structure, determined and refined using X-ray crystallographic analyses (Shrive et al. 1996) and Fourier transform infrared spectroscopy analysis (Dong et al. 1994), assigns pCRP a secondary structure of 50 % β-sheet, 12 % helix, 24 % β turn, and 14 % unordered structure.

When pCRP is dissolved in increasing amounts of urea, it is found to undergo a two-stage structural change as analyzed by Circular dichroism (CD). When urea is increased up to 4 mol/L, pCRP undergoes tertiary structural changes that are consistent with formation of a molten globular structure; secondary structures remain unchanged indicating hydrogen binding forces contributing to helical and beta sheet structures that remain intact. When urea is increased above 4 mol/L, pCRP pentamer dissociates with a substantial loss in secondary structure, shown by a sharp increase in ellipticity at 216 nm (data not shown).

 When urea is removed by dialysis and the mCRP isoform is expressed, some, albeit not as well-organized secondary structure returns, which appears to be predominantly beta structure. C-r_m_CRP CD spectra was similar to that observed for mCRP and was distinct from pCRP (data not shown).

## Discussion

Perhaps the most fundamental biochemistry axiom describing proteins is that structure regulates function. While an understanding of protein structure begins by examining its primary sequence, its secondary, tertiary, and quaternary structures and their dynamic interchange are key elements in deciphering and understanding true biological function. Many proteins exist as precursor molecules, storing critical activation energies in precursor conformations, releasing potential energies when protein structure changes to expose sites needed to drive biochemical or immunological reactions (Dellisanti et al. 2011; Tompa 2012; Cook and Hogg 2013).

Multiple structural forms of CRP have been recognized for years. Hokama et al. (1967) reported that serum CRP was present in 2 forms—a gamma migrating form and a low mobility form. These forms interconverted when mixed with normal serum. Wang et al. (2002) reported that CRP exists in 3 forms—(1) a pentameric ring; (2) a small globulin-like (monomeric) form; and (3) a fibril-like structure. The globulin-like monomers were found on negatively charged membranes in the absence of calcium and were structurally stable. The identity of and interrelationship between these and other structural forms of CRP in various solvents and membrane experimental systems, and their biochemical and immunological characteristics, continue to evolve (Potempa et al. 1983, 1987; Ying et al. 1989, 1992; Zouki et al. 2001; Khreiss et al. 2002, 2004b; Ji et al. 2006a, b; Schwedler et al. 2006; Ji et al. 2007; Wu et al. 2007; Wang et al. 2011). Most fundamentally, CRP appears to exist in two primary structural forms: (1) a serum soluble cyclic pentameric discoid molecule (i.e., pCRP) and (2) a conformationally, antigenically, and functionally distinctive free subunit (i.e., mCRP) which is predominantly tissue-based and poorly soluble.

The widely known and studied cyclic pentamer has been crystalized and its three-dimensional structure published in static form (PDB: 1GNH; 1B09) (Shrive et al. 1996; Thompson et al. 1999; Mikolajek et al. 2011). The concentration of this hepatically synthesized, serum-based protein is a key diagnostic index for the presence of inflammation somewhere in an individual (Kushner 1982; Macintyre et al. 1982; Morley and Kushner 1982; Macintyre et al. 1985; Gewurz et al. 1995; Gabay and Hushner 1999; Vigushin et al. 1993; Black et al. 2004; Marnell et al. 2005; Ma et al. 2013; DuClos 2013; Agrawal et al. 2014). Numerous reports have appeared since 1986, however, showing that CRP is also produced extrahepatically (primarily proven using mRNA hybridization techniques and immunohistochemical analyses), and that antigens related to CRP are widely found not only in serum, but also in various healthy and diseased tissues, including the kidney (Kuta and Baum 1986; Dong and Wright 1996; Yasojima et al. 2001; Calabró et al. 2003; Jabs et al. 2003; Ciubotaru et al. 2005; Sattler et al. 2005; Haider et al. 2006; Krupinski et al. 2006; Wilson et al. 2007; Eisenhardt et al. 2009; Slevin et al. 2010). This tissue-based isoform of CRP is sometimes expressed intracellularly, or tightly bound to membranes, being more characteristic of what is currently known of the mCRP protein. Because both pCRP and mCRP proteins are derived from the same mRNA and because of questions concerning the exact specificity of antibody reagents used to immunochemically identify CRP antigens (Fig. 4; Samberg et al. 1988; Schwedler et al. 2003), many published reports should be interpreted with caution as to which conformational form of CRP was actually being synthesized in various tissue sites.

**Fig. 5:**
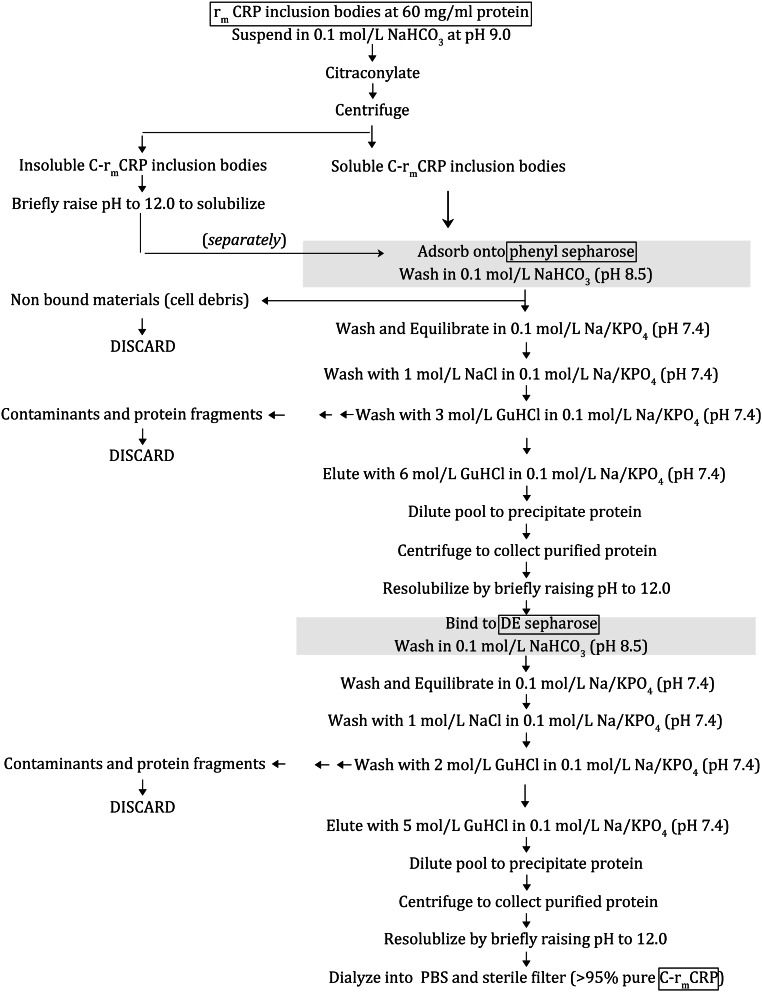
Flow chart for the processing and purification of C-r_m_CRP

Experimental evidence for CRP structural changes has been measured by a loss in intrinsic tryptophan fluorescence and in an increase in exposure of hydrophobic binding sites reactive with the fluorescent dye ANS (Potempa et al. 1983, 1987; Ji et al. 2006a, b, 2007). Additionally, the increased electrophoretic migration of mCRP compared to pCRP (Fig. 3) is an experimental proof that salt-bridge electrostatic charges reported to stabilize the tightly compacted and associated pCRP (Thompson et al. 1999; Agrawal and Chakraborty 2013) are broken when mCRP is expressed. When these intramolecular forces are relaxed, the free electrostatic charges now allow the protein to migrate faster (as an alpha globulin) contrasted to the reported gamma migration for pCRP (Laurent et al. 1983). Of note, literature reports have appeared noting aberrantly migrating CRP in certain disease conditions (Hokama et al. 1967; Hood 1977; Abdulla and Hamadah 1981), suggesting a potential pathological relevance for different isoforms of CRP.

pCRP is known to be stabilized by hydrophobic forces that manifest both at subunit interfaces and in the central core of each of the globular subunits. When mCRP is formed from pCRP, the increased aqueous exposure of these hydrophobic regions promotes mCRP self-aggregation, lowering solubility, and leading to fibril formation (Fig. 1; Wang et al. 2002). The hydrophobic and electrostatic salt-bridges that contribute to the compact tertiary and quaternary structures of pCRP also explain its resistance to proteolysis (Kinoshita et al. 1989); the more relaxed, albeit aggregated mCRP, is found to be much more sensitive to various proteases. Indeed, cleavage of mCRP yields formation of biologically active peptides, such as the C-terminal hexapeptide which has potent anti-inflammatory actions (El Kebir et al. 2011).

As more is learned about the distinctive structures and functions of each CRP isoform, the question arises as to how and where mCRP might be generated and whether mCRP might contaminate pCRP test reagents. Indeed, pCRP has been seen to spontaneous convert into mCRP if stored without calcium or in the presence of chelating agents (Potempa et al. 1987; Wang et al. 2002; Khreiss et al. 2004a). Furthermore, even in the presence of calcium, pCRP converts in a step-wide fashion, into mCRP when incubated for more than 4 h with cells in tissue culture maintained at 37 °C, or with liposomes (Khreiss et al. 2004a; Ji et al. [Bibr CR21][Bibr CR22], 2007). Hence, any extended incubation of pCRP with membranous experimental systems past 4 h may reflect to some extent on the distinctive bioactivities of mCRP. The long history of contradictory reports on CRP may be related to this possibility.

The need for mCRP reagents that can be used for comparative and individualized studies is of paramount importance. Herein, we report on a genetically engineered recombinant form of mCRP (r_m_CRP) and its usefulness as a structural and functional analog of biologically generated mCRP. Selected site-directed mutagenesis was performed to substitute alanine residues for both cysteine residues in the CRP subunit primary sequence, producing a protein that lacks the capacity to form disulfide-linkages. The importance of disulfide reduction as a key regulation mechanism for mCRP binding to lipid raft microdomains has been clearly demonstrated (Wang et al. 2011).

r_m_CRP was expressed in *E. coli* as a highly insoluble aggregated inclusion body, which complicated purification and characterization analyses. We report that acylation of primary amine residues using various anhydride reagents effectively dissociated the aggregates and made the r_m_CRP protein extremely soluble in aqueous solvents. Furthermore, acylation with reagents that reversed the charge of the primary amines did not affect r_m_CRPs hydrophobic binding characteristics, allowing for large scale, efficient use of hydrophobic adsorption chromatography as a first step in purification. The strong, high-yield absorption to this hydrophobic surface allowed various and aggressive washing steps which removed cellular debris and protein fragments, and endotoxin that was present in *E. coli* cellular debris during collection of inclusion bodies. C-r_m_CRP elution from the hydrophobic adsorption column required 6 mol/L Guanidinium HCl. Simple dilution of the guanidinium eluate leads to r_m_CRP precipitation, which allowed for quick recovery, concentration, resolubilization in base, sterile filtration, and facile exchange into chosen buffers for experimental needs. The purification scheme used to purify r_m_CRP is summarized in Fig. 5.

The use of anhydride reagents to alter solubility and activity of various proteins has been reported. Liu and Wang (Liu and Wang 2007) showed modification of chloroperoxidase with maleic anhydride, citraconic anhydride, or phthalic anhydride could improve activity and stability. Shet and Madaiah (1988) showed such chemical modifications could affect immune complex binding, antigenicity, and hemagglutination activity of lectins. Khan and Suriola (1982) showed citraconylation could control the hemagglutination and sugar binding activity of Caster Bean Agglutination. Son et al. (2009) used citraconic anhydride to control the proteolytic activity of trypsin-like enzymes in the recombinant expression, recovery, and yields of proinsulin in *E. coil*.

The biochemical, immunological, and functional activities of C-r_m_CRP were shown to be comparable to biological mCRP, making the recombinant protein an excellent and advantageous research reagent for the study of mCRP. Furthermore, by using citraconylation (in preference to maleylation and succinylation), the acyl groups can be readily removed by short dialysis into citric acid (pH 3.5). Removal of the acyl group does cause r_m_CRP to lose solubility forming fibril-like structures that look more like mCRP than pCRP (Fig. 1C). It is interesting to speculate whether such fibrous-like structures are related to the tissue-deposited mCRP antigen recognized in many studies.

CRP has many homologues in the blood and hemolymph of vertebrates and invertebrates, including the horseshoe crab, which has survived millions of years of evolution (Shrive et al. 1996). In elucidating the true function of CRP in humans and in lower species, one must look beyond its possible role in disease pathology and inflammation, and consider its conceivable role as a key protein of innate immunity, and a necessity factor for general health and survival. As more is learned and appreciated about multiple CRP structures, a reexamination of the role of each isoform of CRP is called for, not only as the prototypic acute phase reactant in reactions of inflammation, but also as an immunological, vascular, and extravascular regulator of health and disease. Indeed, a fundamental biochemical axiom states that protein functions can be regulated by distinct structures.

## Materials and Methods

### Recombinant CRP (r_m_CRP) genetic engineering, expression, and collection as inclusion bodies

CRP is a non-glycosylated protein of five identical subunits, each with a molecular weight of 23 kD and containing one intrachain disulfide bond, arranged in a cyclic discoid structure. Genomic and cDNA clones coding for the human CRP subunit have been isolated (Lei et al. 1985; Woo et al. 1985), and the corresponding protein amino acid sequence has been assigned. The original CRP cDNA clone was isolated from a human liver library and was the kind gift of Drs. Harvey Colton & Bruce Dowton of Harvard University. A number of modification and site-directed mutagenesis procedures were performed on this clone to replace and substitute selected nucleotides for improved, unambiguous expression in *E. coli*, improved plasmid stability, decreased basal transcription, and increased induced expression levels (Reznikoff and Gold 1986). Most specifically, the genetically engineered recombinant CRP subunit (r_m_CRP) was manipulated to purposefully differ from the biological CRP subunit at three residue positions: (1) the N-terminal pyroglutamic amino acid of biological CRP was changed to glutamine and was preceded by a formylated-methionine residue; (2) cysteine residue 36 was substituted with an alanine residue (C36A); and (3) cysteine residue 97 was substituted with an alanine residue (C97A) (collectively known as cys-mutated r_m_CRP) (Potempa et al. 1999).

The Cys-mutated r_m_CRP gene was cloned into the T7 RNA polymerase expression system under the *lacUV5* promoter (Tabor and Richardson 1985), and transformed into *E. coli* BLR(DE3). Transformed bacteria were expanded prior to induction of the r_m_CRP gene with Isopropyl β-D-1-thiogalactopyranoside (IPTG). Induced cells were harvested using continuous flow centrifugation, washed, and suspended in 20 mmol/L Tris HCl, pH 7.6, containing 5 mmol/L EDTA and 1 mmol/L phenylmethylsulfonylfluoride (PMSF) (i.e., “Breakage buffer”) and passed twice through a homogenizer at a pressure of 500 bar. *E. coil* cell pellets were collected, resuspend in buffers, and repassed through the homogenizer at 700 bars. The collected materials were labeled as recombinant mutant CRP subunit Inclusion Bodies (r_m_CR-IBs) and store at below −20 °C until processed.

The plasmid containing the CRP gene pX(T7) CRP13 was isolated from the expression strain and purified on a cesium chloride gradient. The CRP coding region was sequenced by the method of Sanger (Sanger et al. 1977) using ^33^P-dATP and confirmed except for the genetically engineered changes factored into the sequence. No base additions, deletions, or substitutions were unintentionally introduced into the sequence.

### Solubilization of inclusion bodies with anhydride reagents

Citraconic anhydride, maleic anhydride, succinic anhydride, and guanidinium HCl were purchased from Sigma-Aldrich, St. Louis, MO, USA. Phenyl Sepharose (high substitution) and DEAE Sepharose fast flow chromatography resins were purchased from GE Healthcare Life Sciences, Piscataway, NJ, USA. All buffer reagents were analytical grade or better and were purchased from Sigma-Aldrich, VWR (Radnor, PA) or Bio-Rad Laboratories (Hercules, CA).

Chemical modification reactions with anhydride reagents were carried out with slight modifications as described in Means and Feeney (Means and Feeney 1971) and Wong (Wong 2000). Generally, protein suspensions were maintained in 0.1 N Na Bicarbonate buffer at pH 9.0 and small amounts of anhydride reagent (either liquid aliquots with citraconic anhydride or solid crystals for maleic anhydride or succinic anhydride) were added to moderately stirred protein at room temperature. The pH of the mixture was monitored continuously, and drops of NaOH (1 N or 5 N) were added to stabilize and maintain the pH at 9.0. When the pH change was observed to slow or stop, fresh anhydride reagent was added and the process repeated for 5-to-10 cycles. To estimate the level of substitution with maleylation or citraconylation, the change in absorbance at 250 nm was measured, and the moles of substitution per mole of CRP subunit were calculated using an extinction coefficient of *ε* = 3360 (mol/L)^−1^ cm^−1^ (Means and Feeney 1971). Most generally, between 4 and 10 mol of acyl group were quantified for each mole of CRP subunit. Of note, there are 13 lysine residues (the primary site for the acylation reaction with anhydride reagents) in each CRP subunit (Lei et al. 1985; Woo et al. 1985; Thompson et al. 1999).

Part, but not all protein solubilized. The mixture was centrifuged at 3000×*g* at 4 °C for 30 min, and the soluble fraction was separated from the still precipitated protein. The citraconic-solubilized inclusion body (in bicarbonate) was directly processed on Phenyl Sepharose hydrophobic adsorption chromatography resin (see below). The collected precipitated protein was resuspended in 0.1 N NaHCO_3_ and was briefly exposed to pH > 12.0 by addition of 5 N NaOH. After the protein suspension clarified, the sample was quickly returned to pH values of ~8.5 for separate, but identical processing on Phenyl Sepharose.

### Decitraconylation

When desired, citraconylated protein was decitraconylated by dialyzing purified protein in 0.05 mol/L Citrate (pH 3.5) at room temperature for 2 h. Resultant r_m_CRP was insoluble. Maleic groups may also be removed modified protein by acid dialysis. However, removal of maleyl group is incomplete under the described conditions so that use of the 2-methyl maleic anhydride derivative (citraconic anhydride) is the preferred method used herein.

In all purification and processing steps, protein concentrations were determined using a BCA assay (ThermoScientific, Waltham, MA) and by absorbance at 280 nm using Beer’s Law, and a CRP extinction coefficient of 1.95 mg/mL or 44,740 (mol/L)^−1^ cm^−1^.

In the analytical evaluation of various protein samples, Serum Protein Electrophoresis was performed using the Beckman Paragon SPE Gel Electrophoresis System using a precast 1 % Agarose Gel with a barbital running buffer.

### Electron microscopy (EM)

CRP protein samples were diluted to 50~100 μg/mL and observed by negatively stained electron microscopy (Zhu et al. 2011). In brief, 2 μL droplet of sample was added to a freshly glow-discharged carbon-coated 300-mesh copper EM grid for 10 s followed by staining with 1 % phosphotungstic acid (PTA) for 60 s. The grids were observed in a Tecnai G20 (FEI) 200 kV EM.

### Assay for endotoxin

Protein batches were assayed for endotoxin contaminant by Limulus assay (Sigma, St. Louis, MO, USA) according to the instruction. Additional purification step through Detoxi-Gel Columns (Pierce, Rockford, IL, USA) was performed to remove endotoxin when necessary. The final endotoxin level of all protein solutions was below the detection limit 0.06 EU/mL of the assay.

### ELISA analysis for screening monoclonal and polyclonal anti-CRP reagents

Ninety-six well polystyrene microtiter plates were coated with various concentrations of protein, dissolved in 0.1 mol/L Na bicarbonate (pH 9.0). Most routinely, 0.2 μg of protein was coated per well, but for quantitative comparisons of relative specificity of polyclonal reagents for each of native and mCRP, the primary coat protein was varied from 0.001 μg/well to 0.8 μg/well. Because direct coating of the native CRP pentamer onto the polystyrene plate surface results in a loss of native CRP antigenicity, the native protein required calcium-dependent capture to a surface immobilized protein carrier (Keyhole Lympet Hemocyanin (KLH) derivitized with native CRP’s primary ligand –phopshorylcholine (PC) (i.e., PC-KLH).

After backcoating, mouse monoclonal anti-pCRP clone 1D6 or mouse monoclonal anti-mCRP clone 3H12 (Ying et al. 1989, 1992) was added, and bound antibody was detected with goat α-mouse IgG (whole molecule)—peroxidase (Sigma-Aldrich, St Louis, MO) and 2,2′-azino-bis(3-ethylbenzothiazoline-6-sulfonic acid (ABTS) color reagent.

In experiments using polyclonal reagents, pCRP or mCRP antigens were immobilized as described and anti-human CRP—IgG Fraction of Antiserum produced in rabbit (Sigma-Aldrich product C3527—lot 11K9175) was added at various dilutions to immobilized antigens. Bound rabbit antibody was detected with anti-rabbit IgG (whole molecule, developed in goat)-peroxidase conjugate (Sigma-Aldrich Product No. A6667—lot 103K9167) and ABTS color reagent.

The level of reactivity of various dilutions of polyclonal antiserum to normalized levels of protein antigens (0.001 μg/well to 0.8 μg/well) was plotted, and least-squares analysis was used to establish trendlines which were used to compare dilutions needed to detect equivalent reactivity for native and mCRP antigens.

## Abbreviations

CRP: (C-reactive protein)
mCRP: Modified, monomeric C-reactive protein isoform produced when the CRP protein pentamer is dissociated
r_m_CRP: A genetically engineered recombinant analog of mCRP expressed in *E. coli*
C-r_m_CRP: Recombinant mCRP modified with citraconic anhydride
ABTS: Azino-bis(3-ethylbenzothiazoline-6-sulfonic acid
ELISA: Enzyme linked immunosorbent assay
GuHCl: Guanidinium Hydrochloride
mAb: Monoclonal antibody
PC-KLH: Keyhole limpet hemocyanin substituted with phosphoryl choline ligands
SDS-PAGE: Sodium dodecyl sulfate polyacrylamide gel electrophoresis
